# *Notes from the Field*: False-Negative Hepatitis B Surface Antigen Test Results in a Hemodialysis Patient — Nebraska, 2017

**DOI:** 10.15585/mmwr.mm6710a6

**Published:** 2018-03-16

**Authors:** Blake Hendrickson, Saleem Kamili, Tim Timmons, Peter C. Iwen, Caitlin Pedati, Thomas Safranek

**Affiliations:** ^1^Nebraska Department of Health and Human Services, Lincoln, Nebraska; ^2^Laboratory Branch, Division of Viral Hepatitis, CDC; ^3^Lincoln-Lancaster County Health Department, Lincoln, Nebraska; ^4^Nebraska Public Health Laboratory, Omaha, Nebraska.

In March 2017, the Nebraska Department of Health and Human Services (NDHHS) was contacted by a hemodialysis clinic regarding a patient who had tested negative for hepatitis B virus (HBV) surface antigen (HBsAg) after vaccination in 2010 and who later tested positive for HBsAg. A public health investigation subsequently determined that the false-negative results were caused by a surface antigen mutation. Notably, several commercial HBsAg testing kits cannot detect this mutant virus, making it a challenging pathogen for public health surveillance and intervention efforts (*1*).

When the patient started dialysis in 2010, there was no evidence that the patient had ever been tested for HBV. The patient’s vaccination status was also unknown, and 4 doses of HBV vaccine were administered at 0, 1, 2, and 6 months, as recommended for hemodialysis patients (*2*). Postvaccination testing in 2010 was positive for hepatitis B surface antibody (anti-HBs = 82 mIU/mL), indicating immunity to HBV. Postvaccination HBsAg testing performed with a Food and Drug Administration (FDA)–approved assay at Laboratory A was negative. As recommended for patients with demonstrated immunity, only anti-HBs was monitored routinely from 2010 to 2016 to ensure continued protection (anti-HBs >10 mIU/mL) (*2*). In 2016, the patient was hospitalized for acute shortness of breath, and an HBsAg test performed as part of a routine evaluation at laboratory B was positive, indicating a current infection with HBV. The positive result was confirmed by additional testing at commercial and public health laboratories (Table). Insufficient data were available to determine whether the patient acquired HBV infection before or after vaccination.

Specific precautions for HBV-positive patients in a dialysis center include receiving dialysis in a separate room and being assigned separate staff members and equipment (*2*). These control measures were not implemented during 2010–2016 because the index patient had not had a positive HBsAg test result. After the positive HBsAg results were reported, an epidemiologic investigation was initiated by NDHHS and the Lincoln-Lancaster County Health Department to determine the cause of the false-negative test results and to identify any HBV transmission.

A blood sample was collected in April 2017 and sent to the Division of Viral Hepatitis Laboratory Branch at CDC to confirm the HBV diagnosis. CDC testing found high HBV DNA levels (14,200,000 IU/mL), evidence of immunity (anti-HBs = 114 mIU/mL), and HBsAg positivity by one assay and negativity by another assay (Table). Sequencing the S gene of HBV DNA identified an sG145R surface antigen mutation. This mutation is associated with false-negative results, which explains the failure of multiple tests to identify the patient as being HBsAg-positive (*1*).

The epidemiologic investigation identified 45 recent dialysis contacts and 10 close family contacts who were at risk for infection. None of the contacts had prior evidence of HBV infection, all were screened by tests capable of detecting this mutant virus (using a suitable HBsAg assay or by HBV DNA), and all test results were negative, indicating no evidence of HBV transmission, despite the potential exposures to the HBV-infected patient. Family members without evidence of prior HBV vaccination were also advised to complete the HBV vaccination series.

A subsequent survey of laboratories that reported HBsAg results to NDHHS in the previous year identified nine of 23 laboratories using tests that are not known to detect common HBsAg mutations. This included both local hospitals and large national reference laboratories.

The prevalence of sG145R mutations and other HBsAg mutants associated with false-negative test results is not known. However, some studies suggest that mutant strains are found in 6%–12% of chronic HBV carriers (*3*). In addition to issues with diagnostic detection, some mutants are also not recognized and neutralized by protective antibodies induced by current HBV vaccines and HBV immune globulin therapy (*3*).

This case highlights a unique challenge associated with detecting HBV infections when a surface antigen mutation is present. In recent years, some manufacturers have adapted their testing assays to better identify sG145R and other HBsAg mutations. It is important that laboratories use FDA-approved assays that have the ability to detect HBsAg mutants, which was not done in this case at Laboratory A. The clinical and public health community also must be aware of these testing limitations so that discordant results can be identified for correct diagnosis and care of HBV-infected persons and to minimize the spread of these mutant viruses.

**TABLE:** HBsAg lab results for the case patient by facility and testing instrument

Date collected	Laboratory facility	Testing instrument	Result
November 11, 2010	A*	Advia Centaur XPT	Negative
December 8, 2016	A*	Advia Centaur XPT	Negative^†^
December 9, 2016	B*	Advia Centaur XP	Positive
December 14, 2016	C*	Advia Centaur XP	Positive
January 5, 2017	A*	Advia Centaur XPT	Negative^†^
February 2, 2017	A*	Advia Centaur XPT	Negative^†^
March 2, 2017	A*	Advia Centaur XPT	Negative^†^
March 2, 2017	C*	Advia Centaur XP	Positive
May 7, 2017	CDC	Vitros Eci	Negative^†^
May 7, 2017	CDC	Abbott ARCHITECT	Positive
May 23, 2017	NPHL	Advia Centaur XP	Positive
July 25, 2017	D*	ETI-MAK-2 PLUS	Positive
July 25, 2017	E*	Vitros 3600	Negative^†^

**Abbreviation:** NPHL = Nebraska Public Health Lab.

* Deidentified commercial laboratory.

^†^ False-negative result.
