# *Erratum*: Vol. 67, No. 10

**DOI:** 10.15585/mmwr.mm6715a7

**Published:** 2018-04-20

In the report “Notes from the Field: False-Negative Hepatitis B Surface Antigen Test Results in a Hemodialysis Patient — Nebraska, 2017,” in the table on page 312, the testing instrument used by laboratory facility A should have read “**ADVIA Centaur XP.**”

